# Genetic variation in Fcγ receptor IIa and risk of coronary heart disease: negative results from two large independent populations

**DOI:** 10.1186/1471-2350-10-46

**Published:** 2009-05-29

**Authors:** Mahir Karakas, Michael M Hoffmann, Caren Vollmert, Dietrich Rothenbacher, Christa Meisinger, Bernhard Winkelmann, Natalie Khuseyinova, Bernhard O Böhm, Thomas Illig, Winfried März, Wolfgang Koenig

**Affiliations:** 1 Dept. of Internal Medicine II-Cardiology, University of Ulm, Germany; 2 Dept. of Clinical Chemistry, University of Freiburg, Germany; 3 Helmholtz Center Munich, German Research Center for Environmental Health (GmbH), Institute of Epidemiology, Germany; 4 Div. of Clinical Epidemiology and Aging Research, German Cancer Research Center Heidelberg, Germany; 5 Cardiology Group Frankfurt-Sachsenhausen, Frankfurt, Germany; 6 Dept. of Internal Medicine I, University of Ulm, Germany; 7 Synlab, Center of Laboratory Diagnostics, Heidelberg, Germany

## Abstract

**Background:**

The role of the Fcγ receptor IIa (FcγRIIa), a receptor for C-reactive protein (CRP), the classical acute phase protein, in atherosclerosis is not yet clear. We sought to investigate the association of FcγRIIa genotype with risk of coronary heart disease (CHD) in two large population-based samples.

**Methods:**

FcγRIIa-R/H131 polymorphisms were determined in a population of 527 patients with a history of myocardial infarction and 527 age and gender matched controls drawn from a population-based MONICA- Augsburg survey. In the LURIC population, 2227 patients with angiographically proven CHD, defined as having at least one stenosis ≥ 50%, were compared with 1032 individuals with stenosis <50%.

**Results:**

In both populations genotype frequencies of the FcγRIIa gene did not show a significant departure from the Hardy-Weinberg equilibrium. FcγRIIa R(-131) → H genotype was not independently associated with lower risk of CHD after multivariable adjustments, neither in the MONICA population (odds ratio (OR) 1.08; 95% confidence interval (CI) 0.81 to 1.44), nor in LURIC (OR 0.96; 95% CI 0.81 to 1.14).

**Conclusion:**

Our results do not confirm an independent relationship between FcγRIIa genotypes and risk of CHD in these populations.

## Background

Fcγ receptors (FcγR) bind the Fc portion of immunoglobulin (Ig) and thereby link antigen recognition by antibodies with cell-based effector mechanisms [1-4]. Although FcγRIIa (or CD32) [GenBank: BC019931] is expressed on various cells involved in atherogenesis like monocytes, macrophages, platelets and neutrophils, and though it has been detected immunocytochemically in human atherosclerotic lesions, its potential active role in atherosclerosis is still a matter of controversy [5,6]. Calverley et al. showed FcγRIIa expression to be increased in platelets of patients experiencing an acute atherothrombotic event or who were clinically healthy but had two or more atherosclerosis risk factors; by contrast, Pfeiffer et al. reported decreased expression of FcγRIIa on peripheral blood monocytes in patients with severe atherosclerosis [7,8].

A specific genetic polymorphism affecting its function has been described in the FcγRIIa, causing an amino acid exchange from arginine (R) to histidine (H) at position 131. H131 is the only FcγR that recognizes IgG2 efficiently. The codominantly expressed R131 allele has affinity for IgG3 and IgG1 subtypes and exhibits a strongly diminished binding to IgG2 [9].

Increasing evidence suggests that immune processes play a substantial role in atherosclerosis [10]. It has been hypothesized that immune complexes containing immunogenic molecules such as low-density lipoproteins (LDL), heat shock proteins, or microorganisms may activate FcγRIIa within atherosclerotic plaques, subsequently leading to the production and secretion of mediators [11]. On the one hand results from the Rotterdam study have suggested that the IgG subclass specificity of the H131 allele protects against advanced peripheral atherosclerosis. Since the protective effect was independent of traditional cardiovascular risk factors, the authors suggested investigating whether the H131 allele also protects against clinical cardiovascular events [12].

On the other hand, Gavasso et al. assessed the relationship between polymorphisms in three different FcγR genes and coronary artery disease (CAD). In 882 patients undergoing coronary angiography, no association was found between the FcγRIIa genotypes and CAD [13].

FcγRIIa has been identified as the major receptor for human C-reactive protein (CRP) [14]. Pan et al. reported the presence of FcγR's on human EC and their upregulation by cytokines. They described the receptor expression in native EC's being low and getting upregulated by cytokines [15].

CRP, the classical acute phase protein, is a strong cardiovascular risk marker, and responds rapidly to various proinflammatory stimuli [16-18]. Furthermore, several recent studies also suggest a proatherogenic role for CRP. Mechanisms, in support of such a notion include the acceleration of the progression of atherosclerosis in apolipoprotein E-deficient mice, suggesting direct involvement of CRP in atherogenesis [19], and its presence in atherosclerotic lesions but not in the normal vessel wall [20,21].

Devaraj et al. showed that CRP binds to FcγR, mainly FcγRIIa on human aortic endothelial cells (HAEC) to mediate its biological activity, and incubation of HAEC with monoclonal antibodies to FcγRIIa before addition of CRP markedly reversed the proatherogenic effects of CRP on prostacyclin synthase and IL-8. Similar results were observed with intercellular adhesion molecule (ICAM)-1, vascular cell adhesion molecule (VCAM)-1, cGMP and PAI-1 [22]. CRP stimulated matrix metalloproteinase-1 (MMP-1) expression in U937 histiocytes through FcγRII and extracellular signal-regulated kinase (ERK) pathway, suggesting that CRP may be involved in plaque destabilization [23].

Recently, research in this field has focused on the two allelic variants of FcγRIIa. It has been shown that CRP binding is allele-specific. CRP binds with high avidity to monocytes and neutrophils from R131 homozygotes, but shows strongly decreased binding to cells from FcγRIIa H131 homozygotes and intermediate binding to FcγRIIa heterozygotes, implying that the HH131 genotype may thus be less susceptible to coronary heart disease (CHD) [24].

Since there is no direct evidence that the polymorphism may be associated with a lower risk of CHD in vivo we sought to investigate the potential association between the allelic status of FcγRIIa and risk of CHD in two large independent populations.

## Methods

### Study populations

#### MONICA Study

The MONICA (MONItoring of trends and determinants in CArdiovascular disease) Augsburg study was part of the multinational World Health Organisation MONICA project. From 1984/85 to 1994/95, three population-based MONICA surveys had been conducted, and a regional myocardial infarction registry had been established. Altogether 13,427 subjects of caucasian background (6,725 men and 6,702 women) were prospectively followed within the frame of the KORA (Cooperative Research in the Region of Augsburg). The design of the project has been described in detail elsewhere [25]. Five hundred and twenty seven patients with a history of myocardial infarction (MI), identified from the Augsburg Myocardial Register 1996/97, (KORA-B), served as cases, who were compared to 527 age- and gender-matched controls drawn from the population-based MONICA Augsburg survey, conducted in 1994/95.

The presence of CHD was defined as a validated diagnosis of non- fatal and fatal MI according to MONICA diagnostic criteria [26]. Diabetes mellitus (DM) was defined by a positive history, treatment with hypoglycemic drugs or having either random plasma glucose ≥ 200 mg/dl or a HbA1c ≥ 6.5%. Actual hypertension was defined as being aware of hypertension and/or having blood pressure values ≥ 160 mm Hg systolic and/or ≥ 95 mg Hg diastolic. Baseline information on smoking habits, alcohol consumption, medical history, and body mass index (BMI) was gathered by trained medical staff during a standardized interview.

#### LURIC Study

A total of 3,297 patients with suspect CHD participating in the LUdwigshafen RIsk and Cardiovascular Health (LURIC) study were included in these analyses. The design of the study has been described in detail elsewhere [27]. Analyses are based on individuals from whom complete data were available (n = 3,259). Exclusion criteria were incomplete angiographic data, missing data on any of the considered risk factors, acute infectious diseases, or evidence of malignant diseases -possibly associated with an acute phase reaction. All subjects were of caucasian background.

Thus, 2,227 patients with significant CHD were compared with 1,032 individuals with stenoses <50% (control group). DM was diagnosed according to the criteria of the American Diabetes Association (ADA) if plasma glucose was >1.25 g/L in the fasting state or 2.00 g/L two hours after an oral glucose load, which was performed in all subjects not previously diagnosed as having DM, or if individuals received oral hypoglycemic drugs or insulin. Hypertension was defined as having a systolic and/or diastolic blood pressure > 140 and/or 90 mm Hg or having a history of hypertension.

All subjects underwent a standardized interview conducted by trained personnel. The information obtained included current health status -especially physician diagnosed hypertension and DM, medical history, and current medication. Furthermore, sociodemographic characteristics and lifestyle habits were recorded.

Both studies have been approved by the appropriate ethics committees and have therefore been performed in accordance with the Declaration of Helsinki.

### Laboratory methods

#### MONICA Study

A non-fasting venous blood sample was obtained from all study participants while sitting. Total cholesterol was determined with an enzymatic method (CHOD-PAP, Roche Diagnostics, Mannheim, Germany). LDLc was measured after precipitation with dextran sulphate (Quantolip LDL, Immuno AG, Vienna, Austria) and HDLc after precipitation with phosphatungstate acid and Mg^2^^+ ^(Roche Diagnostics, Mannheim, Germany). CRP measurements were done by a high-sensitivity latex enhanced nephelometric assay on a BN II analyser (Dade Behring, Marburg, Germany).

#### LURIC Study

Venous blood was drawn from all participants early in the morning before cardiac catheterization. Total cholesterol was determined enzymatically. HDLc was determined after precipitation with phosphatungstate acid and Mg^2+ ^(Roche Diagnostics, Mannheim, Germany). CRP concentrations were determined using the same high-sensitivity latex-enhanced nephelometric assay on a BN II analyser (N Latex CRP mono, Dade Behring, Marburg, Germany). All laboratory analyses in both studies were done in a blinded fashion.

### Analysis of the R(-131) → H polymorphism of the FcγRIIa gene in MONICA

Genomic DNA has been isolated from white blood cells by standard methods [28]. FcγRIIa genotyping was performed with matrix-assisted laser desorption/ionisation time-of-flight (MALDI-TOF) mass spectrometry [29].

### Analysis of the R(-131) → H polymorphism of the FcγRIIa gene in LURIC

Genomic DNA was prepared from EDTA anticoagulated blood. Receptor polymorphism was determined from polymerase chain reaction and restriction typing. The primers for amplification were 5'-CCTTGGACAGTGATGGTCAC-3' and 5'-TGGAAAATCCCAGAAATTCTCGC-3'. Amplification products were digested with *BstU*I (New England BioLabs) and analysed by agarose gel electrophoresis. The G allele (H131) yielded fragments of 120 and 23 bp; the A allele (R131) was not digested (143 bp).

### Statistical analyses

In both populations demographic, clinical and biochemical characteristics were compared in a descriptive way. Differences in genotype distribution among cases were compared with values predicted by the Hardy-Weinberg equilibrium. The odds ratio (OR) and 95% confidence intervals (CI) for the presence of CHD were assessed by multivariable logistic regression analysis, adjusted for traditional cardiovascular risk factors, given the HH131 genotype compared to RR131 and/or RH131 genotypes. The following established risk factors were used in the fully adjusted models: in MONICA, age, sex, years of education, alcohol consumption, smoking status, BMI, history of hypertension, history of diabetes, HDLc; in LURIC, age, sex, smoking status, BMI, history of hypertension, history of diabetes, and HDLc. Finally, we calculated a power assessment: A two group Chi-square test based on a two-sided α of 0.050 and a power of 80%was used to estimate which statistically significant excess risk (Odds ratio) between cases and control of each study could be detected at minimum based on the proportions of the genotype HH131 versus the other two genotypes combined.

## Results

Table 1 shows demographic characteristics of the MONICA case-control study. The study participants were predominantly men (88.4%). Patients on average had a lower school education, lower alcohol consumption, were more often current- or ex-smokers and also more often had a history of hypertension and DM. BMI and HDLc were similar in both groups. Patients had a geometric mean CRP plasma value of 2.06 mg/L and a mean total cholesterol value of 224 mg/dl. The corresponding levels in controls were 1.49 mg/L for CRP and 241 mg/dl for total cholesterol. The lower level of total cholesterol in patients was due to a more frequent prescription of lipid-lowering drugs.

**Table 1:** Characteristics of Patients with CHD and Controls: MONICA Study

	**CHD patients**	**Controls**
n	527	527
Age, μ (SD)	56.4 (7.1)	56.3 (7.0)
Males (%)	88.4	88.4
School education 9 years or less (%)	66.2	58.8
Average alcohol consumption per day (g) μ (SD)	17.8 (21.7)	26.5 (28.6)
Teetotalers (%)	29.1	19.9
Smoking status (%):		
current smoker	14.6	23.9
ex-smoker	67.6	35.5
never smoker	21.8	40.6
Body mass index (kg/m^2^), μ (SD)	28.4 (3.7)	28.3 (4.0)
History of high blood pressure (%)	54.8	30.7
History of diabetes mellitus (%)	15.8	7.0
Total cholesterol, mg/dl μ (SD)	224.3 (44.4)	240.6 (47.0)
HDL cholesterol, mg/dl μ (SD)	46.9 (14.3)	48.7 (14.3)
CRP, mg/L *	2.1	1.5

* geometric mean; HDL: high-density lipoprotein. CRP: C-reactive protein

Table 2 presents characteristics of participants of the LURIC study. Patients on average were three years older, were more often current- or ex- smokers, and more frequently had a history of hypertension and DM. BMI, HDLc and total cholesterol were similar in both groups. Lipid-lowering drugs, predominantly statins (>97%), were used by 61.2% of CHD patients and by 22.2% of controls. While the average CRP level in patients was 2.86 mg/L (CRP concentration of 1,278 CAD patients with > 50% stenosis with acute coronary syndromes, and unstable angina pectoris were disregarded) this value was 2.55 mg/L in controls.

**Table 2:** Characteristics of Patients with CHD and Controls: LURIC Study

	**CHD patients**	**Controls**
n	2,227	1,032
Age, μ (SD)	63.8 (10.0)	60.2 (11.4)
Males (%)	77.3	53.9
Smoking status (%):		
current smoker	20.6	18.1
ex-smoker	49.8	32.7
never smoker	29.6	49.2
Body mass index (kg/m^2^), μ (SD)	27.5 (3.9)	27.6 (4.4)
History of high blood pressure (%)	54.3	50.6
History of diabetes mellitus (%)	36.6	21.8
Total cholesterol, mg/dl, μ (SD)	189.5 (39.2)	198.7 (37.4)
HDL cholesterol, mg/dl, μ (SD)	37.2 (10.0)	41.9 (11.7)
CRP, mg/L *	3.9 (1.5–9.3)	2.6 (1.1–6.8)

* median and 25^th ^and 75^th ^percentile, respectively;

HDL: high-density lipoprotein. CRP: C-reactive protein

Table 3 shows the genotype frequencies of the FcγRIIa gene among cases and controls. Genotype frequencies in cases and controls, in both populations, did not show a significant departure from the Hardy-Weinberg equilibrium. Furthermore, neither in the MONICA population, nor in the LURIC population a difference among cases and controls with respect to FcγRIIa genotypes existed (p = 0.86 in MONICA and p = 0.64 in LURIC).

**Table 3:** Distribution of FcγRIIa Genotypes and Alleles in CHD Patients and Age- and Gender-Matched Controls in Percent (%): MONICA and LURIC Study

	MONICA*								
	**Men**			**Women**			**All**		
**Genotype**	**Cases** **n = 466**	**Controls** **n = 466**	**P**	**Cases** **n = 61**	**Controls** **n = 61**	**P**	**Cases** **n = 527**	**Controls** **n = 527**	**P**

**RR131**	20.6 (n = 96)	19.5 (n = 91)		19.7 (n = 12)	16.4 (n = 10)		20.5 (n = 108)	19.1 (n = 101)	
**RH131**	48.9 (n = 228)	51.5 (n = 240)		59.0 (n = 36)	47.5 (n = 29)		50.1 (n = 264)	51.0 (n = 269)	
**HH131**	30.5 (n = 142)	28.9 (n = 135)	0.73	21.3 (n = 13)	36.1 (n = 22)	0.19	29.4 (n = 155)	29.8 (n = 157)	0.86
									
**Allele**									
**R**	45.1 (n = 324)	45.3 (n = 331)		49.2 (n = 48)	40.2 (n = 39)		45.5 (n = 372)	44.7 (n = 370)	
**H**	54.9 (n = 370)	54.7 (n = 375)		50.8 (n = 49)	59.8 (n = 51)		54.5 (n = 419)	55.3 (n = 426)	

	LURIC*								

	**Men**			**Women**			**All**		
**Genotype**	**Cases** **n = 1722**	**Controls** **n = 556**	**P**	**Cases** **n = 505**	**Controls** **n = 476**	**P**	**Cases** **n = 2227**	**Controls** **n = 1032**	**P**

**RR131**	21.1 (n = 363)	20.0 (n = 111)		18.4 (n = 93)	19.1 (n = 93)		20.5 (n = 456)	19.6 (n = 202)	
**RH131**	49.1 (n = 846)	47.1 (n = 262)		47.7 (n = 241)	49.4 (n = 235)		48.8 (n = 1087)	48.2 (n = 497)	
**HH131**	29.8 (n = 513)	32.9 (n = 183)	0.38	33.9 (n = 171)	31.5 (n = 150)	0.74	30.7 (n = 684)	32.3 (n = 333)	0.64
									
**Allele**									
**R**	45.6 (n = 1209)	43.5 (n = 373)		42.3 (n = 334)	43.8 (n = 328)		44.9 (n = 1543)	43.7 (n = 699)	
**H**	54.4 (n = 1359)	56.5 (n = 445)		57.7 (n = 412)	56.2 (n = 385)		55.1 (n = 1771)	56.3 (n = 830)	

*The genotype distribution in LURIC as well as in the MONICA study was in Hardy-Weinberg equilibrium among both patients and controls.

Table 4 presents the partially and fully adjusted ORs obtained by logistic regression analysis for the presence of CHD associated with FcγRIIa genotypes. In the MONICA population, the OR for the presence of CHD associated with the HH genotype was 0.92 (95% CI, 0.65 to 1.31) after adjustment for age and gender and increased to 1.01 (95% CI, 0.69 to 1.48) after adjustment for other covariates (Table 4). If the RR and the RH genotype were taken as reference, the OR associated with the HH genotype was 0.99 (95% CI, 0.75 to 1.28) after adjustment for age and gender, and increased to 1.08 (95% CI, 0.81 to 1.44) after additional adjustment for other covariates.

**Table 4:** Partly and Fully Adjusted Odds Ratios for CHD Associated With FcγRIIa Genotypes: MONICA and LURIC Study

**Genotype**	Partly-adjusted OR (95% CI)	Multivariable-adjusted OR (95% CI)
MONICA*		

**RR131**	1 reference	1 reference
**RH131**	0.92 (0.66–1.26)	0.91 (0.65–1.29)
**HH131**	0.92 (0.65–1.31)	1.01 (0.69–1.48)
**RR or RH 131**	1 reference	1 reference
**HH131**	0.99 (0.75–1.28)	1.08 (0.81–1.44)

LURIC**		

**RR131**	1 reference	1 reference
**RH131**	0.95 (0.76–1.18)	0.97 (0.77–1.21)
**HH131**	0.99 (0.81–1.12)	1.01 (0.82–1.25)
**RR or RH 131**	1 reference	1 reference
**HH131**	0.96 (0.81–1.13)	0.96 (0.81–1.14)

*Partly-adjusted for age and gender; Multivariable-adjusted for age, gender, years of education, alcohol consumption, smoking status, BMI, history of hypertension and diabetes, HDL-cholesterol **Partly-adjusted for age and gender; Multivariable-adjusted for age, gender, smoking status, body mass index, history of hypertension, history of diabetes, HDL-cholesterol

In the LURIC population, the OR for CHD associated with the HH genotype in the LURIC population was 0.99 (95% CI, 0.81 to 1.12) after adjustment for age and gender and 1.01 (95% CI, 0.82 to 1.25) after further adjustment for other traditional cardiovascular risk factors. Again, if the RR and RH genotypes were taken as reference, the OR for the association of CHD with the HH genotype was 0.96 (95% CI, 0.81 to 1.13) after partial adjustment for age and gender, and did not change (0.96, 95% CI, 0.81 to 1.14) after multivariable adjustment.

## Discussion

We expected carriers of the HH131 genotype to have a lower risk of CHD because of the strongly decreased binding of CRP to cells expressing the specific FcγIIa receptor. However, results from these two large independent case-control studies do not suggest that the HH131 genotype of FcγRIIa may be associated with a lower risk of CHD.

### Association between FcγRIIa genotype and CHD

Recently published experimental data are in support of the negative findings in our populations. It was shown that CRP binding to monocytes from R131 homozygotes and RH131 heterozygotes was dose-dependent and saturable at approximately 100 mg/L of CRP. The binding avidities of CRP for monocytes from R131 homozygous and RH131 heterozygous donors were 24 and 75 mg/L, respectively [24]. This value is in the range of CRP concentrations present in sera from patients with rather mild to moderate acute phase responses than concentrations postulated in CAD [30]. Since average CRP levels in both populations were less than 4 mg/L, and monocytes from R131 homozygotes bound CRP with an high apparent affinity of 24 mg/L, receptor kinetics do not allow a significant increase in receptor up-regulation. These results are concordant with the observation, that maximum levels of signalling response of FcγRIIa to CRP in differentiated HL-60 granulocytes was recorded at 100–200 mg/L. Thus, one can hypothesize that, although having a lower affinity for the potentially pro-atherogenic CRP, individuals bearing the H131 allele do not benefit, since the concentration of CRP is too low to rise the receptor level in atherosclerotic plaque sufficiently.

As expected, crystal structure analyses showed that the amino-acid position 131 is critical for the interaction with the IgG-Fc-tail [31], and the allelic variants of the FcγR possess distinct phagocytic capacities. The H131 allele is the only FcγR that recognizes IgG2 efficiently, which is the main antibody subtype directed against encapsulated bacteria [9]. In certain conditions gram-negative bacteria may get into the circulation on a daily basis, and thereby stimulate IgG2 production, which plays a pivotal role in phagocytosis of such bacteria [32]. Inflammation, an essential feature in atherosclerosis may be triggered by infectious pathogens [33,34]. It was thought that atherosclerotic cells expressing the HH131 genotype may be less capable for atherosclerosis by being more effective in cleaning IgG2 immune complexes. Several *in vitro *studies demonstrated reduced phagocytosis of IgG2-opsonized particles in cells from individuals homozygous for FcγRIIa-R131 [35]. However, this needs to be discussed critically, as the possibility of CRP-mediated phagocytosis was not taken into consideration sufficiently. CRP binds to encapsulated bacteria like *S. pneumoniae *and *H. influenza*, and maybe in this way provides partial protection from encapsulated infectious pathogens in individuals bearing the R131 allele of FcγRIIa, contributing to a reduction of atherosclerotic risk [24]. The opsonization of *S. pneumoniae*, in the presence of CRP, primarily activates the classical complement pathway and complement is required for the protection by CRP. However, CRP protection does not require FcγR, indicating that CRP is not primarily protective by direct opsonization but by complement and the subsequent opsonophagocytosis [36]. CRP and complement were also shown to be necessary for serum bactericidal activity against *H. influenza*. Using a CRP transgenic mouse model, CRP has also been shown to be protective against gram-negative bacterium [37-39]. This counterbalance of different CRP binding-capacities may affect the contribution of FcγRIIa alleles to host defense and autoimmunity.

In summary, one can hypothesize that there is a critical counterbalance between complement activation by CRP and subsequent protection of bacteria-induced inflammation and cardiovascular disease and the proatherogenic role of CRP in both, the case of strong binding and the case of weak binding of CRP.

Recently three studies suggested the possibility that FcγRI could be the high-affinity receptor for CRP [40-42]. Using ultrasensitive confocal fluorescence microscopy they demonstrated that CRP indeed does bind to FcγRIIa, although with lower affinity than described earlier. Furthermore they studied CRP binding to transfected COS-7 cells expressing the high-affinity IgG receptor FcγRI. They showed that CRP binds to FcγRI on intact cells, with a kd of 10 ± 3 μmol/L. Transfection of COS-7 cells with a plasmid coding for both FcγRI and its functional counterpart, the γ-chain, markedly increased CRP affinity to FcγRI, resulting in a kd of 0.35 ± 0.10 μmol/L. However, results of the recently published studies remain controversial, therefore further research is needed before FcγRI might be established as a high-affinity receptor for CRP.

### Strengths and limitations of our study

Our study has several strengths that need to be mentioned. We carefully selected cases and controls and, in addition, we extensively recorded conventional risk factors in order to assess their potential for confounding and carried out adjustments in multivariate models. The large sample sizes and the independency of the two populations also contribute to the strength of this study. In the MONICA population, all analyses were done in patients with a history of prior MI and compared to population-based controls. In the LURIC population, consecutive cases with angiographically proven stenosis of a major coronary artery of at least 50% were included. Since the FcγRIIa could not be found in mice, such well designed epidemiological studies are of considerable interest in investigating the receptor.

Our study also has some limitations. We used a case-control design in both populations, therefore the temporal relationship of the plasma parameters and disease is difficult to establish. However the temporal relationship of genotype and CHD is certain. Selection and survival bias may represent a further problem. The appropriateness of the control group of the LURIC study deserves discussion. Early lesions undetectable by angiography could not be ruled out in controls, nor was coronary microvascular dysfunction. Nevertheless, coronary angiography remains the gold standard for the diagnosis of clinically relevant CAD, and it is well known that the future incidence of cardiac events is low in subjects with normal coronary angiograms. Although the present study did not have the power to detect a very weak association between FcγRIIa genotypes (i.e. HH131 vs. others) and CHD, it had a power of 80% to detect an OR of 1.46 (α = 0.05) or larger in the MONICA study and an OR of 1.26 or larger in the LURIC study.

## Conclusion

Our results do not suggest an independent and clinically relevant relationship of the FcγRIIa gene polymorphism with risk of CAD. Consequently, it seems unlikely that, in the population studied, FcγRIIa R(-131) → H genotype represents a promising tool for identifying those at increased risk for CHD.

## Abbreviations

AAI: ankle-arm index; ADA: American Diabetes Association; AHA: American Heart Association; BMI: body mass index; CAD: coronary artery disease; CHD: coronary heart disease; CI: confidence interval; CRP: C-reactive protein; DM: diabetes mellitus; EC: endothelial cell; ERK: extracellular signal-regulated kinase; HAEC: human aortic endothelial cell; HDLc: high-density lipoprotein cholesterol; ICAM: intercellular adhesion molecule; Ig: immunoglobulin; IL: interleukin; KORA: Cooperative Research in the Region of Augsburg; LDLc: low-density lipoprotein cholesterol; LURIC: LUdwigshafen RIsk and Cardiovascular Health; MI: myocardial infarction; MMP: matrix metalloproteinase; MONICA: MOnitoring trends and determinants in CArdiovascular disease; OR: odds ratio; PAI: plasminogen activator inhibitor; TNF: tumour necrosis factor; VCAM: vascular cell adhesion molecule.

## Competing interests

The authors declare that they have no competing interests.

## Authors' contributions

MK drafted the manuscript. TI, CV, WM and MHH carried out molecular genetic studies, participated in the study design and coordination. DR and WM analysed the data. BB, CM, BW and NK contributed to sample collection and critically revised the manuscript. WK conceived the study and critically revised the manuscript. All authors read and approved the final manuscript.

## Pre-publication history

The pre-publication history for this paper can be accessed here:


